# Supplementary material for: GenTB: A user-friendly genome-based predictor for tuberculosis resistance powered by machine learning
Source: Genome Med. 2021 Aug 30;13:138. doi: 10.1186/s13073-021-00953-4 (PMC8407037; doi:10.1186/s13073-021-00953-4)
Supplement: Supplementary file 2 — Additional file 2: Fig S1. Characteristics of variables used for resistance prediction by Gentb-RF for isoniazid and rifampicin. Fig S2. Probability distribution and selected threshold for Gentb-RF resistance predictions. Fig S3. Probability distribution and thresholds according to [17] of Gentb-WDNN resistance predictions. Fig S4. User-friendliness evaluation of the GenTB tool. Fig S5. Diagnostic performance of the four prediction tools across antituberculosis drugs. Fig S6. Sequencing depth of resistance-conferring genes in isolates falsely predicted susceptible to first line agents. Fig S7. Sequencing depth of resistance-conferring genes in isolates falsely predicted susceptible to second line agents. Table S1. Genetic loci used for random forest model training. Table S2. Phenotypic drug susceptibility testing methods used by studies included in this benchmarking dataset. Table S3. Frequencies and percentages of available drug susceptibility data per drug. Table S4. Diagnostic accuracy comparison of tools for drugs with insufficient phenotype data and pyrazinamide performance on all isolates. Table S5. Area under the Receiver Operating Characteristic curve for GenTB-RF and GenTB-WDNN. Table S6. Diagnostic accuracy to rifampicin and isoniazid across low-depth and passed-depth isolates. Table S7. Non-silent variants in the gene rpoB among isolates with discordant phenotype and genotype predictions for the drug rifampicin. Table S8. Non-silent variants in the genes inhA, katG, ahpC, or fabG1 among isolates with discordant phenotype and genotype predictions for the drug isoniazid. [file 13073_2021_953_MOESM2_ESM.docx]

**GenTB: A user-friendly genome-based predictor for**

**tuberculosis resistance powered by machine learning**

ADDITIONAL FILE 2

**Authors:**

Matthias I Gröschel^1^, Martin Owens^1^, Luca Freschi^1^, Roger Vargas Jr^1,2^, Maximilian G Marin^1,2^, Jody Phelan^3^, Zamin Iqbal^4^, Avika Dixit^1,5^ and Maha R Farhat^1,6^

**Affiliations**

^1^ Department of Biomedical Informatics, Harvard Medical School, Boston, MA, USA

^2^ Department of Systems Biology, Harvard Medical School, Boston, MA, USA

^3^ Faculty of Infectious and Tropical Diseases, London School of Hygiene & Tropical Medicine, London WC1E 7HT, UK

^4^ European Bioinformatics Institute, Hinxton, Cambridge CB10 ISD, UK

^5^ Division of Infectious Diseases, Boston Children’s Hospital, Boston, MA, USA

^6^ Division of Pulmonary and Critical Care Medicine, Massachusetts General Hospital, Boston, MA, USA

**
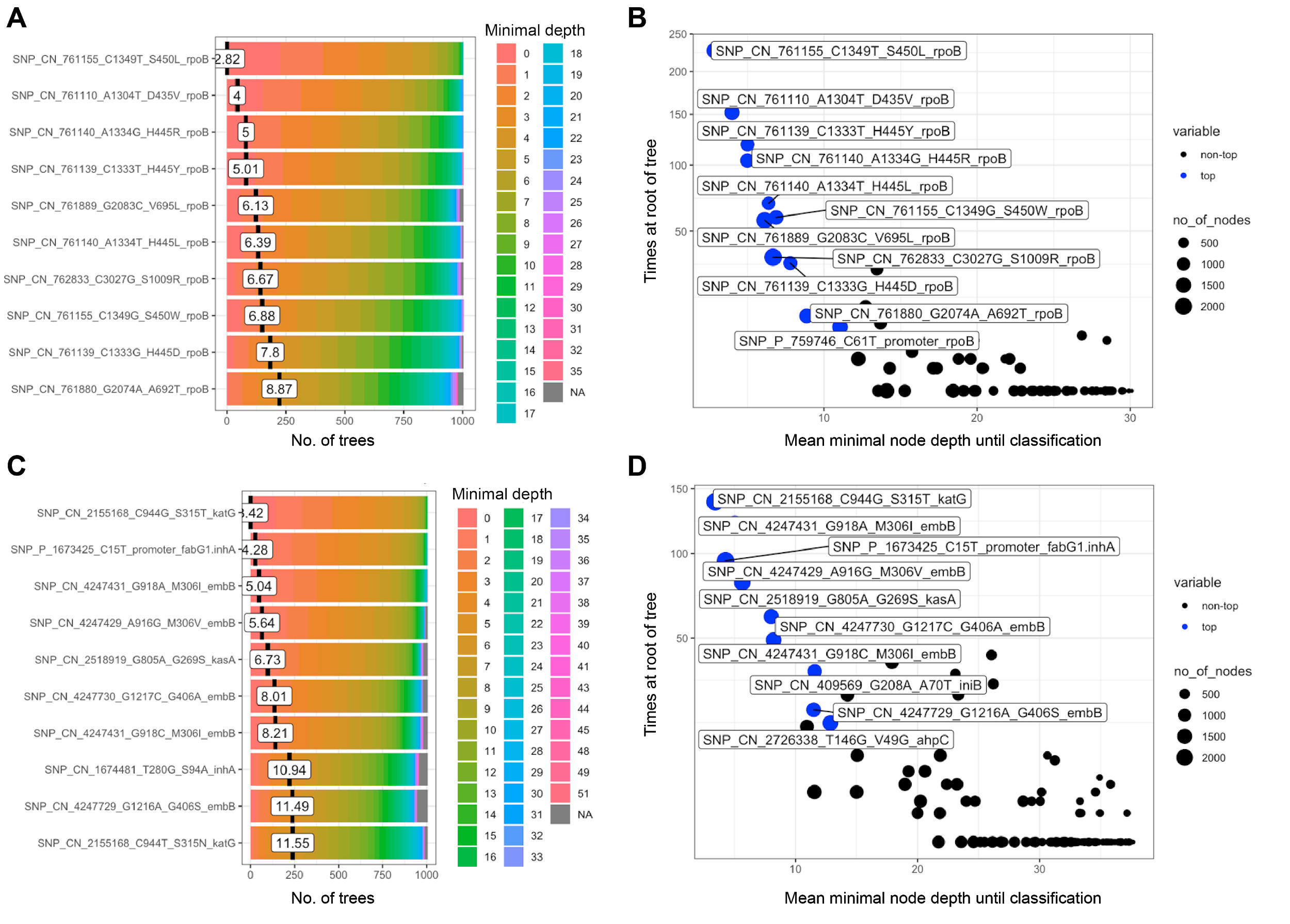
**

**Fig S1: Characteristics of variables used for resistance prediction by Gentb-RF for isoniazid and rifampicin. A)** Distribution of minimal node depth among the trees of the classification forest for rifampicin resistance is shown. The mean of the distribution is marked by a vertical bar with a value label on it, denoting the mean number of node depth required for the variant to reach classification into resistant or susceptible. **B)** Multi-way importance for rifampicin resistance classification showing mean depth of first split on the variant on the X-axis, the number of trees where the variable is at the root of the tree on the y-axis, and the total number of nodes in the forest that split on that variant by size of the dots. **C)** and **D)** as **A)** and **B)** for isoniazid resistance classification. Genetic variants are described as follows and are separated by underscores: First type of variant, second if the variant leads to change in amino acid (AA), frameshift, or stop codon, third the genomic coordinate based on the reference strain H37RV (AL123456), fourth AA change, fifth codon change, last locus tag.

**
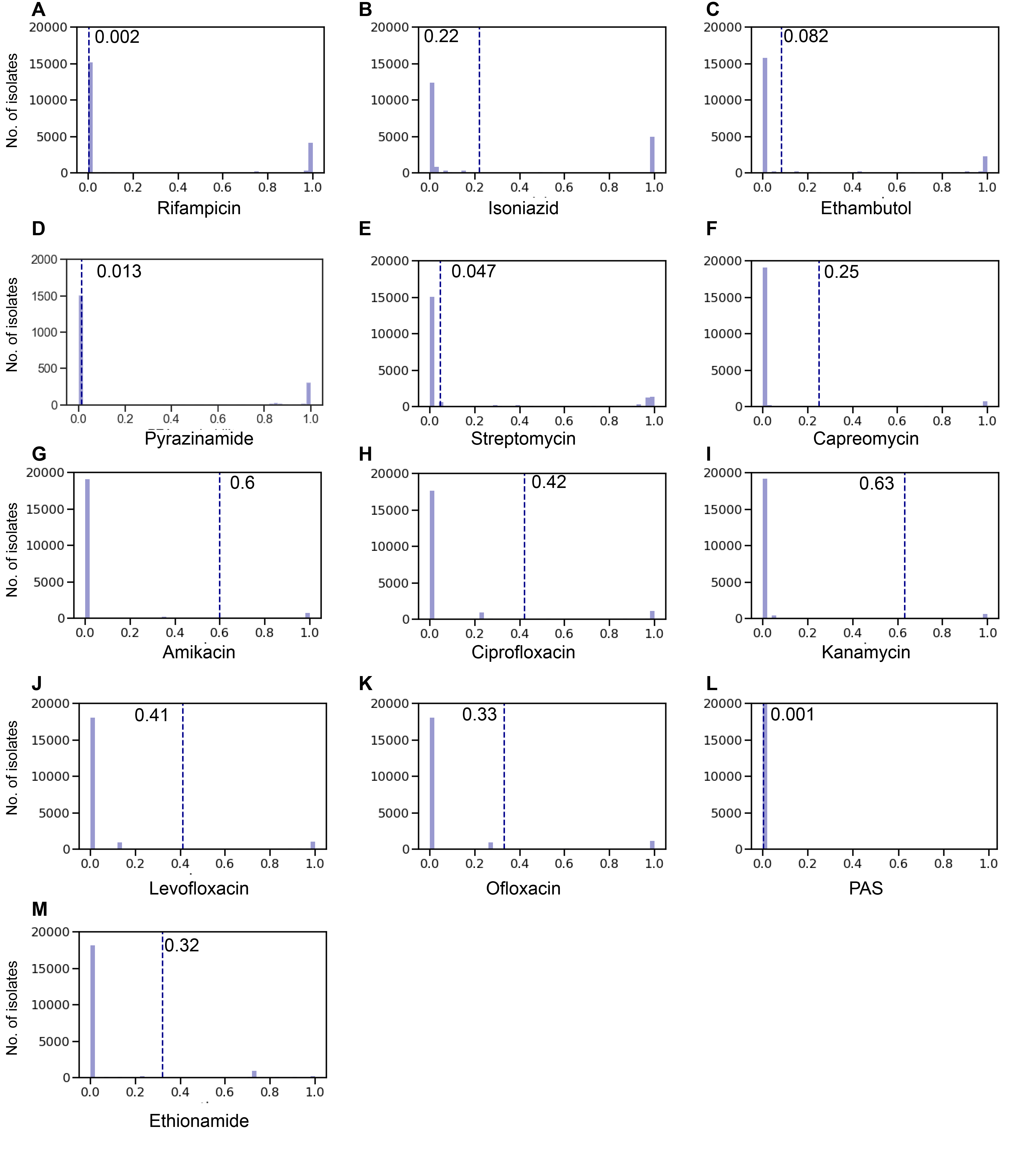
 Fig S2: Probability distribution and selected threshold for Gentb-RF resistance predictions.** A-M. Probability of susceptibility (0 = drug susceptibility, 1 = drug resistance) for 13 drugs among the 20.379 isolates as predicted by Gentb-RF. Vertical lines depict the resistance threshold that yielded the highest predictive performance as measured by the sum of sensitivity and specificity. Drug-specific probability values are written in each subpanel.

**
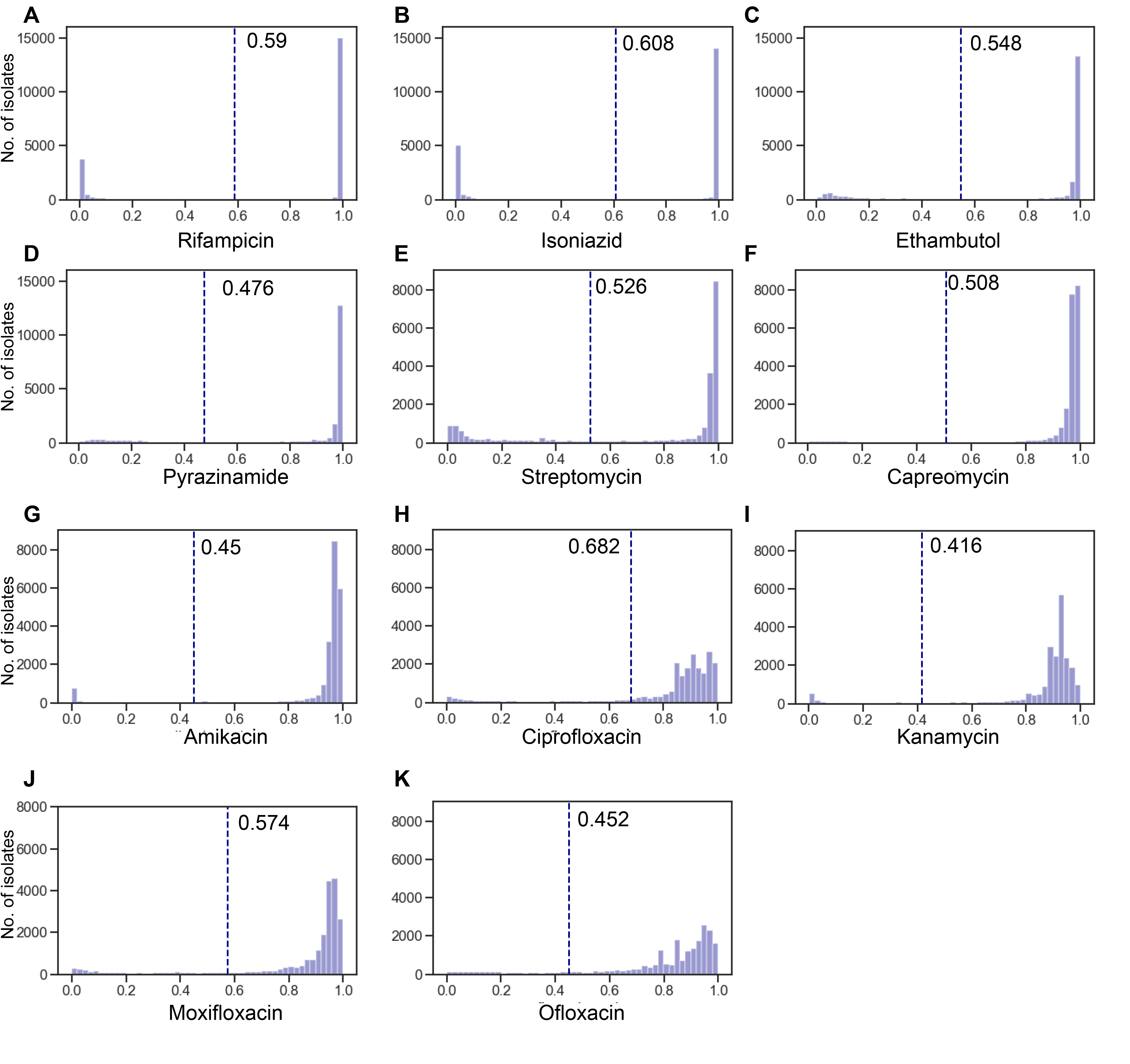
 Fig S3: Probability distribution and thresholds according to** [1] **of Gentb-WDNN resistance predictions.** A-K. Probability of susceptibility (1 = drug susceptibility, 0 = drug resistance) for 11 drugs among the 20.379 isolates as predicted by Gentb-WDNN. Vertical lines depict the resistance threshold that yielded the highest predictive performance as measured by the sum of sensitivity and specificity. Drug-specific probability values are written in each subpanel.

***
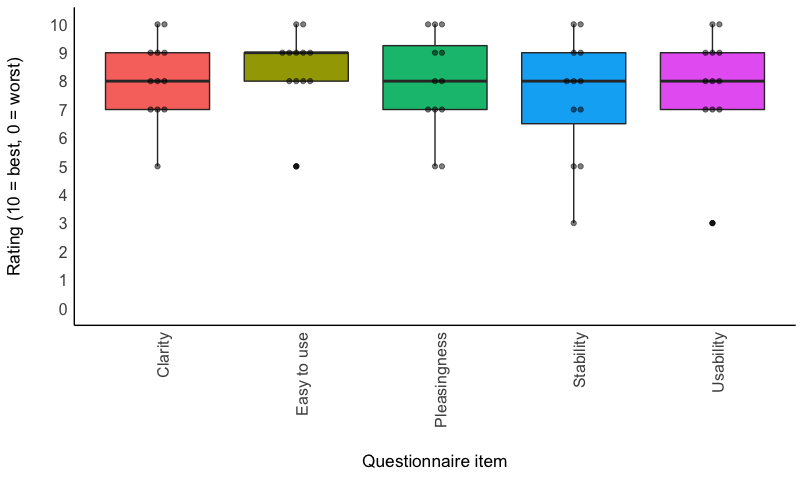
*
Fig S4: User-friendliness evaluation of the GenTB tool.** Box plots displaying user’s responses on a scale from 1 (worst) to 10 (best) across five questionnaire items.


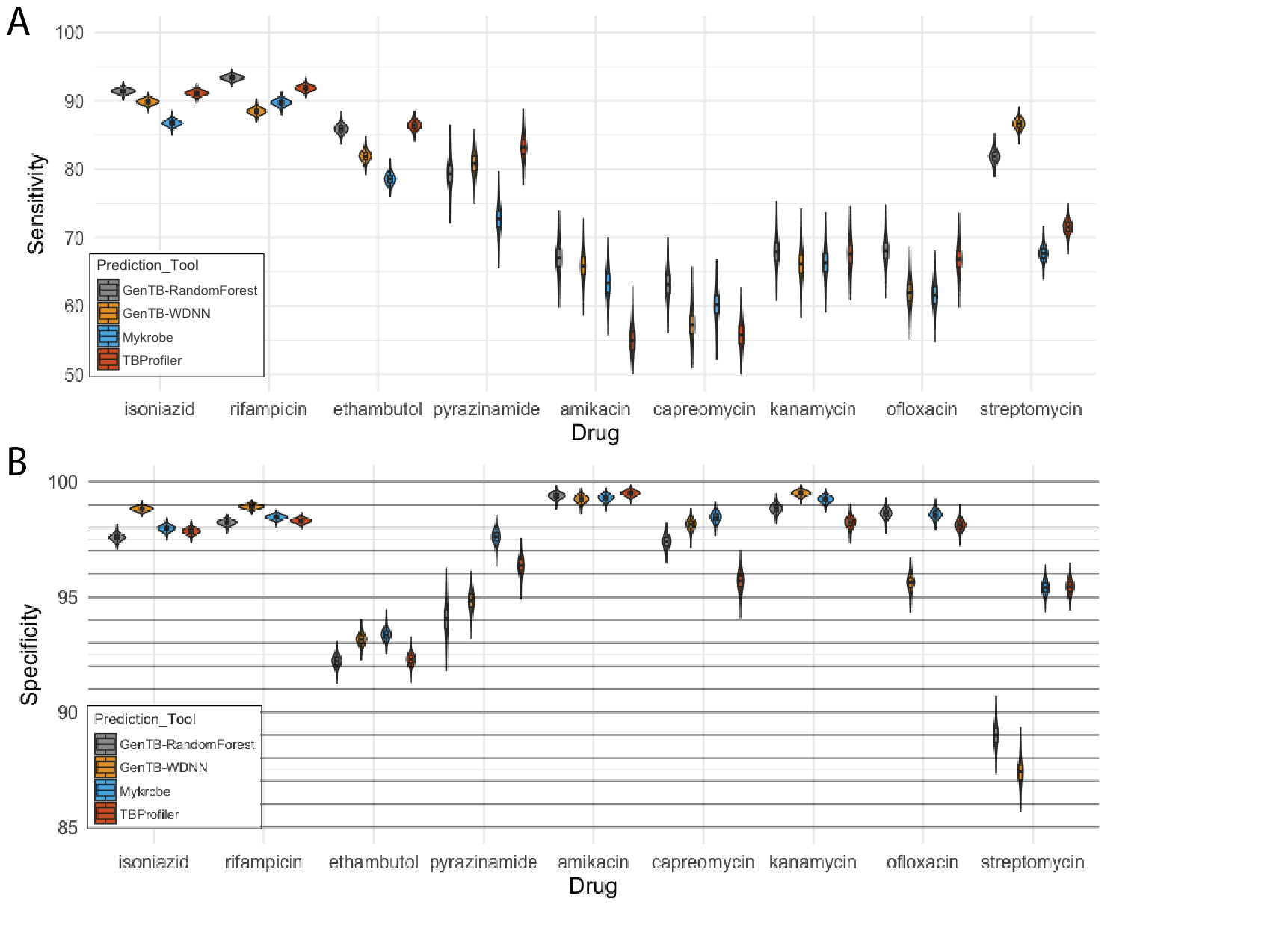


**Fig S5: Diagnostic performance of the four prediction tools across antituberculosis drugs.** Violin plots displaying **A)** sensitivity and **B)** specificity of the used prediction tools to diagnose drug resistance by drug.

**
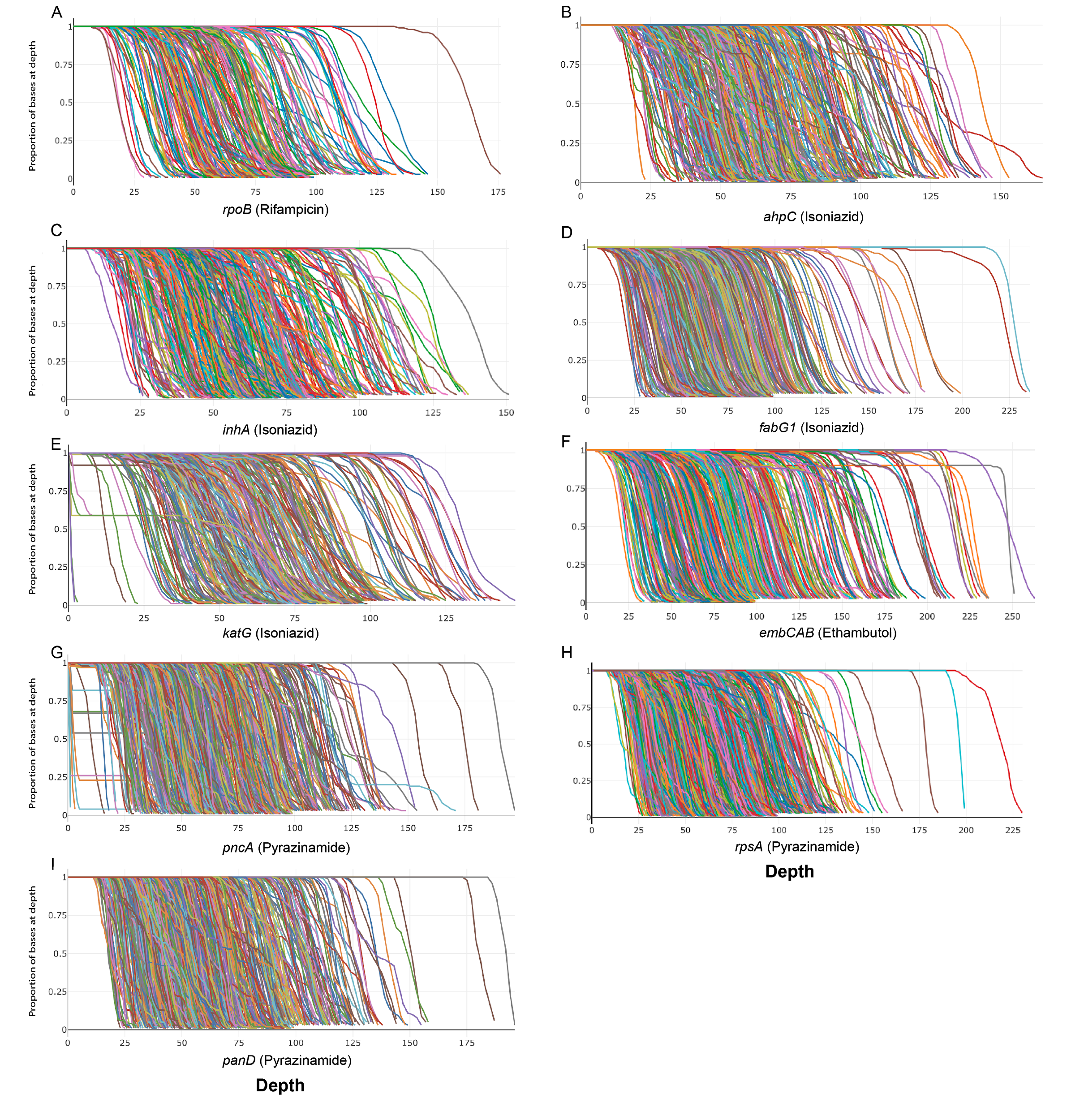
 Fig S6: Sequencing depth of resistance conferring genes in isolates falsely predicted susceptible to first line agents.** The plots **A)** to **I)** display the sequencing depth against the proportions of bases covered at this depth for the length of the respective genes. Each line represents one isolate that was predicted susceptible by GenTB-RF while phenotypically resistant.

**
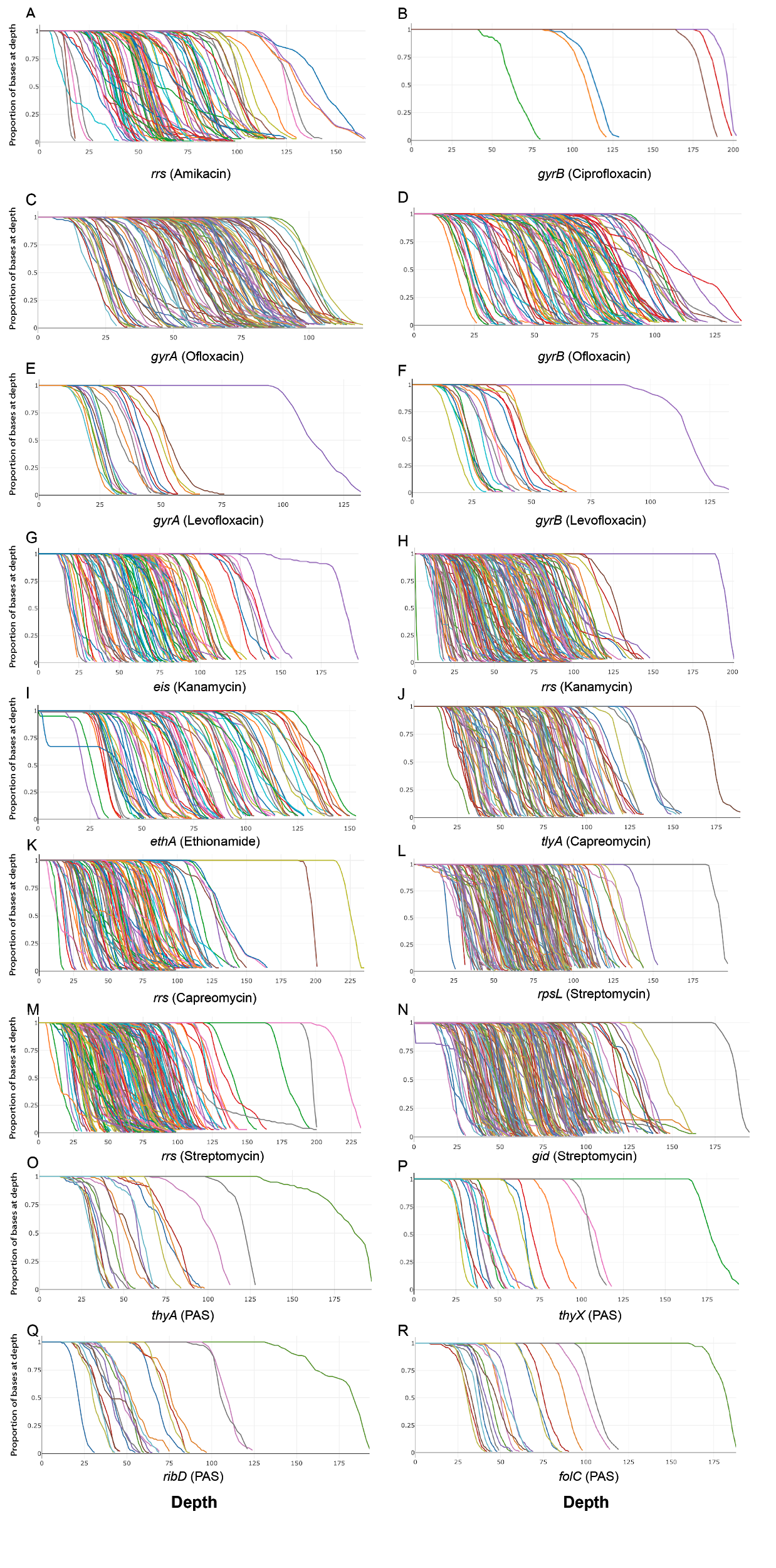
**

**Fig S7: Sequencing depth of resistance conferring genes in isolates falsely predicted susceptible to second line agents.** The plots **A) to R)** display the sequencing depth against the proportions of bases covered at this depth for the length of the respective resistance genes noted below each plot. Each line represents one isolate that was predicted susceptible by GenTB-RF while phenotypically resistant.

**Table S1: Genetic loci used for random forest model training**

|  | Prediction model | |
| --- | --- | --- |
| Drug | GenTB-RF [2] | GenTB-WDNN [1] |
| Isoniazid | *katG, inhA* (+ promoter), *fabG1, embB, kasA,  ahpC* (+ promoter), *oxyR, iniA, iniB, iniC, ndh* | *katG, inhA* (+ promoter), *fabG1, embB, kasA,  ahpC* (+ promoter), *oxyR, iniA, iniB, iniC, ndh* |
| Rifampicin | *rpoB* | *rpoB* |
| Ethambutol | *embB, embA, embC, iniA, iniB, iniC* | *embB, embA, embC, iniA, iniB, iniC* |
| Pyrazinamide | *pncA* | *pncA, rpsA* (+ promoter) |
| Streptomycin | *rpsL, rrs, gid* | *rpsL, gid* |
| Ethionamide | *ethA, inhA* (+ promoter) | - |
| Ciprofloxacin | *gyrA, gyrB* | *gyrA, gyrB* |
| Ofloxacin | *gyrA, gyrB* | *gyrA, gyrB* |
| Levofloxacin / Moxifloxacin | *gyrA, gyrB* | *gyrA, gyrB* |
| Amikacin | *rrs, rrl* | *rrs, rrl* |
| Capreomycin | *rrs, rrl, tlyA* | *rrs, rrl, tlyA* |
| Kanamycin | *rrs, rrl* | *rrs, rrl, eis* (+ promoter) |
| Para-aminosalicylic acid | *thyA* | - |

**Table S2:** Phenotypic drug susceptibility testing methods used by studies included in this benchmarking dataset.

| **Data Source** | **Phenotypic Drug Susceptibility Method** |
| --- | --- |
| PATRIC (https://www.patricbrc.org/) | Various |
| ReSeqTB (https://platform.reseqtb.org/) | Various |
| CRYPTIC [3] | BACTEC Mycobacterial Growth Indicator Tube (MGIT) 960 system (Becton Dickinson), by culture on 7H10 or Löwenstein–Jensen (LJ) agar, or by microscopic-observation drug-susceptibility (MODS) assay |
| Farhat et al. [4] | First line drugs using LJ proportion method, pyrazinamide using Wayne method, second line using 7H11 agar proportion method |
| Walker et al. [5] | MGIT 960, LJ or resistance ratio method |
| Casali et al. [6] | MGIT 960 for first and second line, pyrazinamide – MGIT or semisolid medium |
| Coll et al. [7] | BACTEC 460 TB System (Becton Dickinson), MGIT 960 system, solid agar or LJ slopes |
| Hicks et al. [8] | Minimum inhibitory concentrations (MICs) measured using the Alamar Blue reduction assay after resuspension in 7H12 media |
| Guerra-Assucao et al. [9] | Not reported |
| Wollenberg et al. [10] | LJ medium (BACTEC MGIT PZA for Pyrazinamide) |
| Dheda et al. [11] | Sensititre MYCOTB MIC plates or MGIT 960 |
| Zignol et al. [12] | LJ proportion method or MGIT 960 |
| Phelan et al. [13] | Not reported |
| Klopper et al. [14] | MGIT 960 |
| Phelan et al. [15] | First line drugs using the proportion method. Second line drugs PAS was tested on LJ at 0.5 μg/ml. The other drugs were tested on Middlebrook 7H11 agar. |

**Table S3:** Frequencies and percentages of available drug susceptibility data per drug

| **Drug name** | **Resistant** | | **Susceptible** | | **Unknown** | |
| --- | --- | --- | --- | --- | --- | --- |
|  | *n* | % | *n* | % | *n* | % |
| amikacin | 623 | 3.1 | 3,563 | 17.5 | 16,193 | 79.5 |
| capreomycin | 652 | 3.2 | 3,846 | 18.9 | 15,881 | 77.9 |
| ciprofloxacin | 63 | 0.3 | 331 | 1.6 | 19,985 | 98.1 |
| ethambutol | 3,001 | 14.7 | 12,788 | 62.8 | 4,590 | 22.5 |
| ethionamide | 502 | 2.5 | 1,095 | 5.4 | 18,782 | 92.2 |
| isoniazid | 6,141 | 30.1 | 13,509 | 66.3 | 729 | 3.6 |
| kanamycin | 583 | 2.9 | 3,878 | 19.0 | 15,918 | 78.1 |
| levofloxacin | 111 | 0.5 | 69 | 0.3 | 20,199 | 99.1 |
| moxifloxacin | 426 | 2.1 | 4,149 | 20.4 | 15,804 | 77.6 |
| ofloxacin | 762 | 3.7 | 4,313 | 21.2 | 15,304 | 75.1 |
| para.aminosalicylic_acid | 46 | 0.2 | 478 | 2.3 | 19,855 | 97.4 |
| pyrazinamide | 2,374 | 11.6 | 12,199 | 59.9 | 5,806 | 28.5 |
| rifampicin | 5,155 | 25.3 | 14,885 | 73.0 | 339 | 1.7 |
| streptomycin | 2,150 | 10.6 | 5,012 | 24.6 | 13,217 | 64.9 |
| **MDR & XDR** | | | | | | |
| MDR | 4,743 | 23.3 | - | - | - | - |
| XDR | 396 | 1.9 | - | - | - | - |
| Note: MDR = Multi drug-resistant, XDR = Extensively drug-resistant. | | | | | | |

**Table S4:** Diagnostic accuracy comparison of tools for drugs with insufficient phenotype data and pyrazinamide performance on all isolates

| **Drug** | **Phenotype** | | **GenTB - RF** | | **GenTB - WDNN** | | **Mykrobe** | | **TB-Profiler** | |
| --- | --- | --- | --- | --- | --- | --- | --- | --- | --- | --- |
|  |  | | **Isolates sequenced with high depth (n = 19.880)** | | | | | | | |
|  | **R (n)** | **S (n)** | **Sensitivity (95% CI)** | **Specificity (95% CI)** | **Sensitivity (95% CI)** | **Specificity (95% CI)** | **Sensitivity (95% CI)** | **Specificity (95% CI)** | **Sensitivity (95% CI)** | **Specificity (95% CI)** |
| ciprofloxacin | 63 | 330 | 78% (66 to 88) | 98% (97 to 99) | 93% (85 to 100) | 97% (95 to 99) | 66% (53 to 78) | 98% (97 to 100) | 90% (83 to 97) | 98% (97 to 100) |
| levofloxacin | 65 | 104 | 81% (73 to 88) | 77% (66 - 87) | - | - | - | - | 74% (65 to 83) | 75% (64 to 86) |
| para-aminosalicylic_acid | 46 | 474 | 9% (2 to 18) | 100% (99 to 100) | - | - | - | - | 30% (17 to 44) | 98% (96 to 99) |
| pyrazinamide | 2,336 | 11,932 | 90% (88 to 91) | 88% (87 to 90) | 81% (79 to 82) | 95% (94 to 95) | 72% (71 to 74) | 98% (97 to 98) | 81% (80 to 83) | 96% (96 to 97) |

Note: Tool's performance on all isolates with available pyrazinamide phenotype shown, for performance on the hold-out validation dataset after Random Forest retraining please refer to Table 1.

**Table S5:** Area under the Receiver Operating Characteristic curve for GenTB-RF and GenTB-WDNN

| **Drug** | **GenTB-RF** | **GenTB-WDNN** |
| --- | --- | --- |
|  | Area under the ROC curve (95% CI) | |
| ciprofloxacin | 0.88 (0.82 to 0.93) | 0.95 (0.91 to 0.99) |
| levofloxacin | 0.79 (0.72 to 0.85) | - |
| para-aminosalicylic_acid | 0.54 (0.51 to 0.59) | - |

RF = Random Forest, WDNN = Wide and Deep Neural Network

**Table S6:** Diagnostic accuracy to rifampicin and isoniazid across low-depth and passed-depth isolates.

| Tool | Drug | Low-depth isolates (n = 499) | | Passed-depth isolates  (n = 19,880) | |
| --- | --- | --- | --- | --- | --- |
|  |  | Mean Sensitivity (SD) | Mean Specificity (SD) | Mean Sensitivity (SD) | Mean Specificity (SD) |
| GenTB-RF | isoniazid | 84.6 (3.64) | 98.2 (0.66) | 91 (0.36) | 97.6 (0.13) |
| GenTB-RF | rifampicin | 87.3 (3.64) | 98.5 (0.59) | 93.4 (0.37) | 98 (0.1) |
| GenTB-WDNN | isoniazid | 83.7 (3.77) | 99.4 (0.36) | 89.9 (0.4) | 98.9 (0.09) |
| GenTB-WDNN | rifampicin | 81.5 (4.19) | 99 (0.48) | 88.5 (0.45) | 98.9 (0.09) |
| TBProfiler | isoniazid | 75.6 (4.3) | 98.5 (0.61) | 91.1 (0.37) | 97.9 (0.13) |
| TBProfiler | rifampicin | 82.7 (4.05) | 98.8 (0.54) | 91.8 (0.40) | 98.3 (0.1) |
| Mykrobe | isoniazid | 70.4 (4.53) | 98.7 (0.56) | 86.7 (0.44) | 97.9 (0.13) |
| Mykrobe | rifampicin | 76.9 (4.49) | 98.7 (0.54) | 89.7 (0.44) | 98.5 (0.1) |

Note: GenTB-RF = GenTB Random Forest, GenTB-WDNN = GenTB - Wide and Deep Neural Network, SD = Standard Deviation

**Table S7**: Non-silent variants in the gene *rpoB* among isolates with discordant phenotype and genotype predictions for the drug rifampicin

| False negative predictions by GenTB-RandomForest  (*n* = 333 isolates) | |  | False positive predictions by GentTB-RandomForest  (*n* = 254 isolates) | |
| --- | --- | --- | --- | --- |
| Variant | count |  | variant | count |
| INS_CI_761103_i1296TTC_433F_rpoB^¶^ | 14 |  | SNP_CN_761155_C1349T_S450L_rpoB^¶^ | 49 |
| INS_CI_761135_i1328GAC_443L_rpoB^¶^ | 9 |  | SNP_CN_761095_T1289C_L430P_rpoB^¶^ | 33 |
| SNP_CN_761101_A1295T_Q432L_rpoB^¶^ | 9 |  | SNP_CN_761139_C1333A_H445N_rpoB^¶^ | 31 |
| DEL_CD_761101_d1294AATTCATGG_432_rpoB^¶^ | 8 |  | SNP_CN_761889_G2083C_V695L_rpoB | 30 |
| DEL_CD_761115_d1308AAC_437_rpoB^¶^ | 6 |  | SNP_CN_761109_G1303T_D435Y_rpoB^¶^ | 30 |
| SNP_CN_760555_A749G_E250G_rpoB | 5 |  | SNP_CN_761277_A1471T_I491F_rpoB | 29 |
| DEL_CF_763258_d3451G_1151_rpoB | 4 |  | SNP_CN_761161_T1355C_L452P_rpoB^¶^ | 26 |
| DEL_CD_761105_d1298CATGGA_433_rpoB^¶^ | 3 |  | SNP_CN_761110_A1304T_D435V_rpoB^¶^ | 9 |
| DEL_CD_761083_d1276GCACCA_426_rpoB^¶^ | 2 |  | SNP_CN_761139_C1333T_H445Y_rpoB^¶^ | 5 |
| DEL_CF_762516_d2709G_904_rpoB | 2 |  | SNP_CN_761155_C1349G_S450W_rpoB^¶^ | 5 |
| INS_CI_761099_i1292CCA_431S_rpoB^¶^ | 2 |  | SNP_CN_761139_C1333G_H445D_rpoB^¶^ | 4 |
| SNP_CN_761141_C1335A_H445Q_rpoB^¶^ | 2 |  | SNP_CN_761167_C1361T_P454L_rpoB | 3 |
| DEL_CD_761069_d1262CAAGGAGTTCTTCGGCAC_421_rpoB | 2 |  | SNP_CN_761110_A1304G_D435G_rpoB^¶^ | 3 |
| DEL_CD_761100_d1293CAA_432_rpoB^¶^ | 2 |  | SNP_CN_761140_A1334T_H445L_rpoB^¶^ | 2 |
| DEL_CD_761088_d1281AGCCAGCTG_428_rpoB^¶^ | 2 |  | SNP_CN_761880_G2074A_A692T_rpoB | 2 |

Note: the 15 most frequent variants or variant combinations are shown. Variants are denoted as follows: First type of variant, second if the variant leads to change in amino acid (AA), frameshift, or stop codon, third the genomic coordinate based on the reference strain H37RV (AL123456), fourth AA change, fifth codon change, last locus tag. ^¶^ Variant located in the rifampicin resistance determining region (RRDR)

**Table S8**: Non-silent variants in the genes *inhA, katG, ahpC,* or *fabG1* among isolates with discordant phenotype and genotype predictions for the drug isoniazid

| False negative predictions by GenTB-RandomForest  (*n* = 518 isolates) | |  | False positive predictions by GentTB-RandomForest  (*n* = 315 isolates) | |
| --- | --- | --- | --- | --- |
| Variant | count |  | Variant | count |
| SNP_CN_2155129_C983A_W328L_katG | 10 |  | SNP_CN_2155168_C944G_S315T_katG^† ¶^ | 56 |
| SNP_CN_2154016_C2096T_G699E_katG | 6 |  | SNP_CN_1674481_T280G_S94A_inhA^† ¶^ | 14 |
| SNP_CN_2155689_C423G_L141F_katG | 5 |  | SNP_CN_1674782_T581C_I194T_inhA^†^ | 10 |
| SNP_CN_2155786_G326A_A109V_katG^†^ | 5 |  | SNP_CN_2154695_C1417G_V473L_katG | 10 |
| SNP_CN_2154661_C1451T_R484H_katG | 4 |  | SNP_CN_2154075_C2037G_Q679H_katG | 2 |
| SNP_CN_2155665_C447G_W149C_katG | 3 |  | SNP_CZ_2154077_G2035A_Q679*_katG | 2 |
| SNP_CN_2155690_A422G_L141S_katG | 3 |  | SNP_CN_2726338_T146G_V49G_ahpC^†^ ^¶^ | 2 |
| SNP_CN_1674782_T581C_I194T_inhA^†^ | 3 |  | SNP_CN_1674263_T62C_I21T_inhA^†^ | 2 |
| SNP_CN_2155102_T1010C_Y337C_katG^†^ | 3 |  | SNP_CN_2155168_C944T_S315N_katG^†^ ^¶^ | 2 |
| SNP_CN_1674262_A61G_I21V_inhA^†^ | 3 |  | SNP_CN_2154676_G1436A_A479V_katG | 1 |
| SNP_CN_2154641_C1471T_G491S_katG | 3 |  | DEL_CF_2154510_d1602C_535_katG | 1 |
| SNP_CN_2154688_G1424A_T475I_katG | 3 |  | SNP_CN_2726323_C131G_P44R_ahpC^†^ | 1 |
| SNP_CN_2155819_T293C_Y98C_katG | 3 |  | SNP_CN_2155258_C854G_G285A_katG | 1 |
| SNP_CN_2155222_C890A_G297V_katG | 3 |  | SNP_CN_2154760_C1352T_G451D_katG | 1 |
| SNP_CN_2154730_T1382G_Q461P_katG | 3 |  | SNP_CN_2155648_T464C_Y155C_katG^†^ | 1 |

Note: The 15 most frequent variants are shown; Known lineage markers are excluded. ^†^ Variants that GenTB-RF has seen before. ^¶^ Variant considered important for isoniazid resistance by GenTB-Random Forest. We excluded variants in genes *kasA* and *embB* as their role in isoniazid resistance is questioned.

**SUPPLEMENT REFERENCES**

1. Chen ML, Doddi A, Royer J, Freschi L, Schito M, Ezewudo M, et al. Beyond multidrug resistance: Leveraging rare variants with machine and statistical learning models in Mycobacterium tuberculosis resistance prediction. EBioMedicine. 2019;43:356–69.

2. Farhat MR, Sultana R, Iartchouk O, Bozeman S, Galagan J, Sisk P, et al. Genetic Determinants of Drug Resistance in Mycobacterium tuberculosis and Their Diagnostic Value. Am J Respir Crit Care Med. 2016;194:621–30.

3. CRyPTIC Consortium and the 100,000 Genomes Project, Allix-Béguec C, Arandjelovic I, Bi L, Beckert P, Bonnet M, et al. Prediction of Susceptibility to First-Line Tuberculosis Drugs by DNA Sequencing. N Engl J Med. 2018;379:1403–15.

4. Farhat MR, Freschi L, Calderon R, Ioerger T, Snyder M, Meehan CJ, et al. GWAS for quantitative resistance phenotypes in Mycobacterium tuberculosis reveals resistance genes and regulatory regions. Nat Commun. 2019;10:2128.

5. Walker TM, Kohl TA, Omar SV, Hedge J, Del Ojo Elias C, Bradley P, et al. Whole-genome sequencing for prediction of Mycobacterium tuberculosis drug susceptibility and resistance: a retrospective cohort study. Lancet Infect Dis. Elsevier BV; 2015;15:1193–202.

6. Casali N, Nikolayevskyy V, Balabanova Y, Ignatyeva O, Kontsevaya I, Harris SR, et al. Microevolution of extensively drug-resistant tuberculosis in Russia. Genome Res. 2012;22:735–45.

7. Coll F, Phelan J, Hill-Cawthorne GA, Nair MB, Mallard K, Ali S, et al. Genome-wide analysis of multi- and extensively drug-resistant Mycobacterium tuberculosis. Nat Genet. 2018;50:307–16.

8. Hicks ND, Yang J, Zhang X, Zhao B, Grad YH, Liu L, et al. Clinically prevalent mutations in Mycobacterium tuberculosis alter propionate metabolism and mediate multidrug tolerance. Nat Microbiol. 2018;3:1032–42.

9. Guerra-Assunção JA, Crampin AC, Houben RMGJ, Mzembe T, Mallard K, Coll F, et al. Large-scale whole genome sequencing of M. tuberculosis provides insights into transmission in a high prevalence area. Elife [Internet]. 2015;4. Available from: http://dx.doi.org/10.7554/eLife.05166

10. Wollenberg KR, Desjardins CA, Zalutskaya A, Slodovnikova V, Oler AJ, Quiñones M, et al. Whole-genome sequencing of Mycobacterium tuberculosis provides insight into the evolution and genetic composition of drug-resistant tuberculosis in Belarus. J Clin Microbiol. 2017;55:457–69.

11. Dheda K, Gumbo T, Maartens G, Dooley KE, McNerney R, Murray M, et al. The epidemiology, pathogenesis, transmission, diagnosis, and management of multidrug-resistant, extensively drug-resistant, and incurable tuberculosis. The Lancet Respiratory Medicine. 2017;5:291–360.

12. Zignol M, Cabibbe AM, Dean AS, Glaziou P, Alikhanova N, Ama C, et al. Genetic sequencing for surveillance of drug resistance in tuberculosis in highly endemic countries: a multi-country population-based surveillance study. Lancet Infect Dis. Elsevier BV; 2018;18:675–83.

13. Phelan JE, Lim DR, Mitarai S, de Sessions PF, Tujan MAA, Reyes LT, et al. Mycobacterium tuberculosis whole genome sequencing provides insights into the Manila strain and drug-resistance mutations in the Philippines. Sci Rep. Springer Science and Business Media LLC; 2019;9:9305.

14. Klopper M, Heupink TH, Hill-Cawthorne G, Streicher EM, Dippenaar A, de Vos M, et al. A landscape of genomic alterations at the root of a near-untreatable tuberculosis epidemic. BMC Med. Springer Science and Business Media LLC; 2020;18:24.

15. Phelan J, Coll F, McNerney R, Ascher DB, Pires DEV, Furnham N, et al. Mycobacterium tuberculosis whole genome sequencing and protein structure modelling provides insights into anti-tuberculosis drug resistance. BMC Med [Internet]. Springer Science and Business Media LLC; 2016;14. Available from: http://dx.doi.org/10.1186/s12916-016-0575-9
