# Supplementary material for: GenTB: A user-friendly genome-based predictor for tuberculosis resistance powered by machine learning
Source: Genome Med. 2021 Aug 30;13:138. doi: 10.1186/s13073-021-00953-4 (PMC8407037; doi:10.1186/s13073-021-00953-4)
Supplement: Supplementary file 3 — Additional file 3. Questionnaire to evaluate the user-friendliness of the GenTB tool. [file 13073_2021_953_MOESM3_ESM.pdf]

GenTB: A user-friendly genome-based predictor for tuberculosis resistance powered by machine learning

Authors:

Matthias I Gröschel[1], Martin Owens[1], Luca Freschi[1], Roger Vargas Jr[1,2], Maximilian G Marin[1,2], Jody Phelan[3], Zamin Iqbal[4], Avika Dixit[1,5] and Maha R Farhat[1,6]

ADDITIONAL FILE 3

Questionnaire to evaluate the user-friendliness of the GenTB tool

User friendliness of the GenTB tool

As previous GenTB users we would like to solicit your help to evaluate how easy it is to use GenTB.

OPTION A: Only answer the questions below without running a prediction now.

OPTION B: Run a antibiotic resistance prediction based on provided FASTQ files and report on your experience using the questions below. To do this, please follow these tasks:

1) Create or login to your user account on https://gentb.hms.harvard.edu/

2) Click on 'Predict' and then on 'Create a prediction from a set of pair-ended FastQ genetic sequences', and provide any dataset name and description.

3) Dropbox link to FASTQ files:
https://www.dropbox.com/sh/tnvw8cg1fh0ag0w/AAAu0wk5Dwt6FwfqlwoGYv7ya?dl=0

3a) Download the FASTQ files and upload from your computer, or

3b) Download the FASTQ files to your personal Dropbox, and upload from Dropbox (in this case we need to send you a dropbox invite, please let us know).

We appreciate your feedback and would love to hear back by next Monday, May 10th, 2021.

Many thanks!

Matthias, Martin and Maha

1. How pleasing did you find the GeTB site?

Mark only one oval.

	1	2	3	4	5	6	7	8	9	10	
Not pleasing at all											Very pleasing

2. How clear was the information on GenTB?

Mark only one oval.

	1	2	3	4	5	6	7	8	9	10	
Not clear at all	⬭	⬭	⬭	⬭	⬭	⬭	⬭	⬭	⬭	⬭	Very clear

3. How easy to use is GenTB?

Mark only one oval.

	1	2	3	4	5	6	7	8	9	10	
Not easy at all	⬭	⬭	⬭	⬭	⬭	⬭	⬭	⬭	⬭	⬭	Very easy

4. How stable was GenTB during your evaluation?

Mark only one oval.

	1	2	3	4	5	6	7	8	9	10	
Not stable at all	⬭	⬭	⬭	⬭	⬭	⬭	⬭	⬭	⬭	⬭	Very stable

5. How usable do you find GenTB?

Mark only one oval.

	1	2	3	4	5	6	7	8	9	10	
Not usable at all	⬭	⬭	⬭	⬭	⬭	⬭	⬭	⬭	⬭	⬭	Very usable

6. Do you have any other feedback?

This content is neither created nor endorsed by Google.

Google Forms
